# Applying Digital Twins to Research the Relationship Between Urban Expansion and Vegetation Coverage: A Case Study of Natural Preserve

**DOI:** 10.3389/fpls.2022.840471

**Published:** 2022-02-15

**Authors:** Dongmiao Zhao, Xuefei Li, Xingtian Wang, Xiang Shen, Weijun Gao

**Affiliations:** ^1^Innovation Institute for Sustainable Maritime Architecture Research and Technology (iSMART), Qingdao University of Technology, Qingdao, China; ^2^School of Environmental and Municipal Engineering, Qingdao University of Technology, Qingdao, China; ^3^Department of Statistic, George Washington University, Washington, DC, United States; ^4^Faculty of Environmental Engineering, The University of Kitakyushu, Kitakyushu, Japan

**Keywords:** vegetation research, urbanization, digital twins, remote sensing, convolution neural network

## Abstract

With the growth of the world population, cities expand and encroach on forests and plants, causing many environmental problems. Digital Twin, as the rapidly developing technique in recent years, provides the opportunity to implement the specific situation of forests and plants at present or in the future, which has great performance on predictive analysis and optimization. From the consideration of plants and forests, this study provides a comprehensive case study to research the relationship between urban development boundary and natural environment in a natural preserve in a coastal city. Multispectral data of the study area is collected by Unmanned Aerial Vehicle (UAV), combining satellite remote sensing (RS) historical data and geographic data to establish the digital twin model for plant identification. In conjunction with local Master planning of land use, the results of modeling are used to analyze the influences of urban construction on the natural environment, and the inappropriate aspects of the planning are discovered and summarized. In addition, 6 suggestions for effective management and planning strategies are presented. As plants and forests are effective factors of natural conditions, this study offered an objective assessment for the sustainability and rationality of urban planning with some guidance and bases.

## 1. Introduction

One of the most significant issues in urbanization is developing in a friendly way with biodiversity conservation (Marzluff, 2002; Mcdonnell and Hahs, 2013). To benefit city dwellers and establish a stable urban green system, city planners always make efforts on the diverse conservation and the sustainability of ecology (Rieke et al., 2014; Nilon et al., 2017; Gja et al., 2019). However, the natural environment around cities is facing the threat of degeneration. A total of 0.18–0.32 million hectares of forest have been lost worldwide caused by cropland displacement since these croplands are occupied by urban expansion (van Vliet, 2019). It is the urban sprawl that should be responsible for a large amount of loss of natural habitat. In the past three decades, the area of natural habitat has dropped all over the world and are threatened by increasing potential risks (Abila, 2020). Even though rapid urbanization has a significant promotion on economic development, it has become a detrimental factor of environmental sustainability (Grimm et al., 2008). To improve urban and natural environmental sustainability, it is essential to study and understand the effects of urban expansion on the natural environment better. The more important is to figure out appropriate strategies and effective management methods to promote the sustainability of urbanization.

As a rapidly developing technique in recent years, the digital twin has been used in a wide range of domains, such as energy (Sivalingam, 2018), automotive (Caputo et al., 2019), and agriculture (Pylianidis et al., 2021). The digital twin has shown its remarkable performance in addressing complex problems. It uses real-world data of a physical object or system and represents them in a dynamic virtual presentation across multiple processes of its lifecycle combined simulation and machine learning with data analysis. The digital twin is able to understand, learn, and reason what-if questions in intuitive approaches. Remote sensing (RS) data are processed comprehensively and generate valuable intelligence for various research areas, such as the prediction of land cover change Boulila et al. (2017); Ferchichi et al. (2017), disaster damage detection Vetrivel et al. (2017). With the increasingly adopted technique of digital twins, RS provides the opportunity to implement the specific situation of forests and plants at present, and to summarize the characteristics of historical changes.

This study aims to research the relationship between urban expansion and the natural environment for the last 15 years by analyzing a comprehensive case study from the consideration of plants and forests. The research area is part of a natural preserve in a coastal city of China since the conflict between urban construction and natural ecology conservation is quite intense. The multispectral data of the research area are collected by Landsat series satellite and Unmanned Aerial Vehicle (UAV). To identify different vegetation types in the research area, two regular RS indexes are employed in this study, which are Normalized Difference Vegetation Index (NDVI) and Green Normalized Difference Vegetation Index (GNDVI). Moreover, based on field investigation data, Convolutional Neural Network (CNN) modeling is implemented and compared with traditional index methods. The identification results can support the analysis on spatial-temporal characteristics, thus providing the methodology of problem discovery and decision support. The major contributions of this study are expressed as follows:

1. Combined with deep learning techniques, this study provides a high-accuracy CNN model to identify types of plants from multispectral images collected by UAVs. Besides, it makes a comparison of performance on plant identification between indexes and the CNN model.
2. Offer spatial-temporal analysis of research area and summarize the characteristics of land cover change and current problems under the circumstance of urbanization for the last 15 years. In addition, 6 appropriate suggestions are supplied for future urban expansion in this area.
3. This study combined the technique of digital twins in the whole research process, from data collection, data modeling, visualization, and decision support, providing a good reference case for related research.

The rest of this study is organized as follows: Section 2 presents the related work on the effects of urban sprawl and related techniques that have been used in this study; Section 3 mainly introduces the methodology, concluding introduction of the research area, data collection process, main indexes used for analysis; and Section 4 provides the experiment result with temporal and spatial analysis, besides, discussion and suggestion for future urban construction; and Section 5 is the conclusion of this study.

## 2. Related Studies

It cannot be ignored that urban sprawl is responsible for the majority degradation of natural and agricultural land (Sp et al., 2019). Various studies have analyzed the impacts of urban sprawl on the natural environment. Wetland biodiversity in the Front Range region in America was estimated under the circumstance of urban expansion (Pieter et al., 2012). Under the effect of urban sprawl, the loss of natural habitat was simulated by Yanmin et al. (2019). Yang et al. (2020) took Changbai Mountain as a research area to assess and analyze the potential loss of natural habitat until 2,050 caused by urbanization. The impairment of environmental sustainability was assessed by analyzing the patterns and modes of urban expansion (Liu et al., 2019). Plants, as the largest cover on earth and the most important factor affecting the human living environment, have always been the core content of ecological research. Vegetation is an appropriate medium to study the interaction between nature and cities. The influences of urbanization on forests in Perth were assessed by Ramalho et al. (2014). By presenting a green infrastructure proposal, Capotorti et al. restored and reconnected urban vegetation with the delivery of regulating services (Capotorti et al., 2017). Ji et al. (2020) analyzed the unbalanced forest displacement in three Chinese coastal urban groups from 1992 to 2015, finding the negative effects on forest-coverage quantity and landscape quality.

Remote sensing technology has been widely used in the ecological domain. Nowosad et al. summarized and assessed the global spatial distribution land coverage changes based on annual global land cover maps from the period of 1992 to 2015 (Nowosad et al., 2018). Forest coverage and landscape pattern changes in Chinese inland and coastal urban groups were explored from the RS landcover dataset (Zhu et al., 2019). Applied to the Landsat archive, the information of annual land use and coverage between 1985 and 2017 for Brazil were reconstructed as automatic mapping (Souza et al., 2020). In recent years, UAVs have been widely used in acquiring aerial accurate maps, since the operation of their sensors has been improved with high-performance (Hamylton et al., 2020). Along with the rapid development of UAV flight control technologies (Kose and Oktay, 2020; Oktay, 2021), it has been used in thermal image acquisition for wildlife detection, classification, and conservation (Luis et al., 2016). UAV with multispectral cameras has high potential in mapping submerged marine fauna (Colefax et al., 2018). Environmental landscapes have been better studied and managed through applications of vegetation RS by UAVs, e.g., identifying the coverage degree of ground vegetation (Ghazal et al., 2015), mapping and monitoring non-submerged aquatic plants (Husson et al., 2016), classifying vegetation communities in the wetland (Fu et al., 2021), and estimate biomass of grass (Niko et al., 2018). As UAV technologies and associated methodologies have been improved with advanced development, they are more affordable and have been increasingly adopted in many research domains.

One of the most widespread vegetation indexes is the NDVI (Tucker, 1979; Tucker et al., 2005), which is data from satellite imagery to reflect the growth condition of plants. NDVI is essential in the studies of ecology. Li et al. developed a new method to monitor the real-time growth of crops by using NDVI percentiles (Li et al., 2019). In the Three Gorges Reservoir Region, China, NDVI was used to reflect the impacts of local climate, population density, urbanization, and other social factors on vegetation (Wen et al., 2017).

Machine learning and cloud computing techniques are rapidly developed in recent years (Zhang et al., 2020), and many researchers apply traditional and modified modeling methods to process RS data. Chai focused on cloud and cloud shadow detection in Landsat data and proposed an approach based on deep CNNs (Chai et al., 2019). Han employed CNN in NWPU VHR-10 satellite data for target recognition and achieve great performance (Han et al., 2020), and Yi proposed a novel approach based on probabilistic faster R-CNN for object detection (Yi et al., 2021). Chen presented DRSNet (Chen and Tsou, 2021), an architecture for image scene classification in low-resolution data, and Swain implemented dimensionality reduction techniques to hyperspectral data (Swain and Banerjee, 2021). Moreover, De Lima researched transfer learning in CNN-based classification in RS data (Pires de Lima and Marfurt, 2020). These studies provide the foundation of research and prove the feasibility of this study.

## 3. Methodology

**Figure 1:**
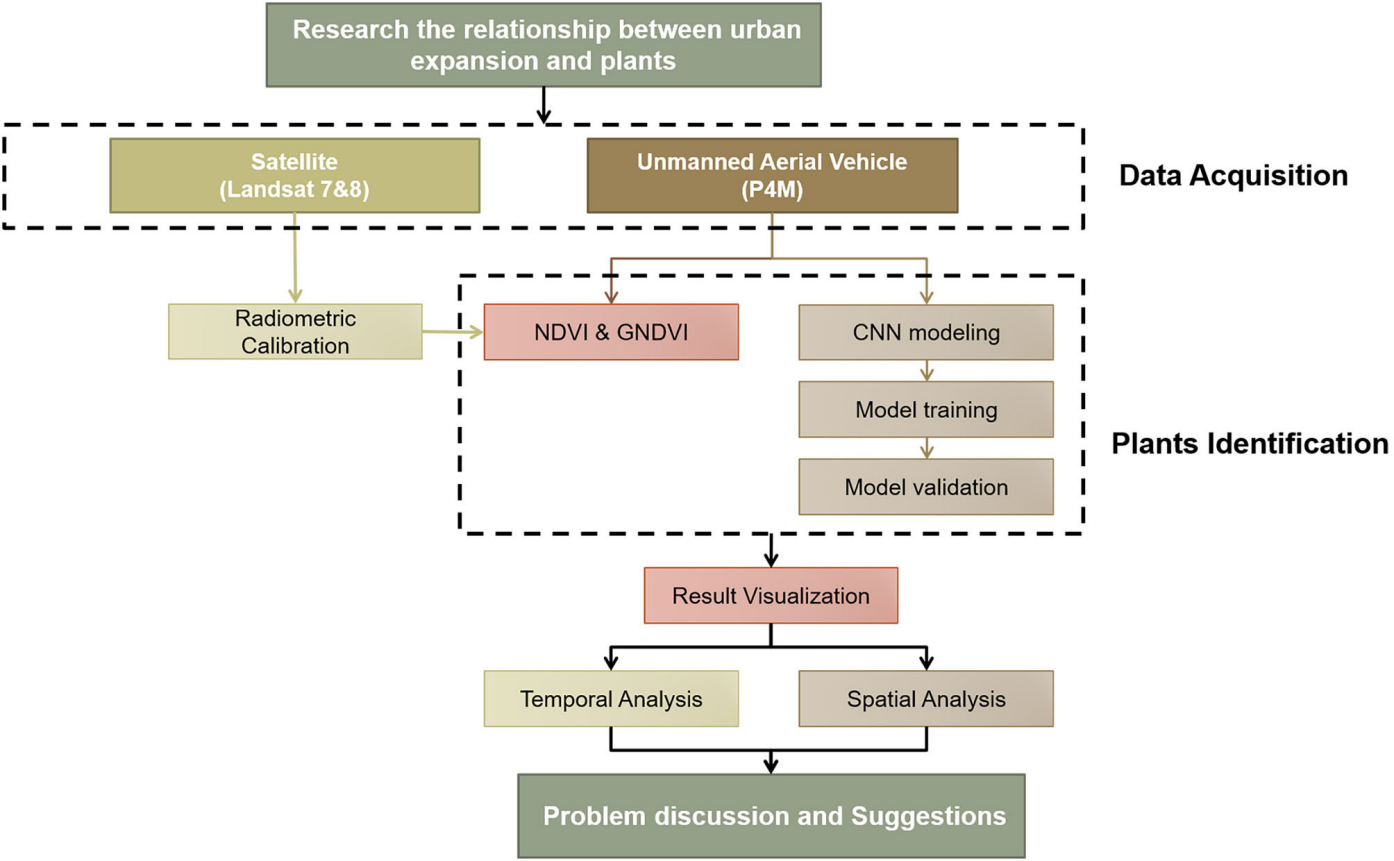
The process of research.

To research the spatial-temporal characteristic and transition of plants in the research area, this study employs various sensing techniques to acquire data from the real world, and apply advanced data modeling to process the collected data to represent physical entities in cyberspace. Figure 1 describes the major procedures of this study.

### 3.1. Research Area

**Figure 2:**
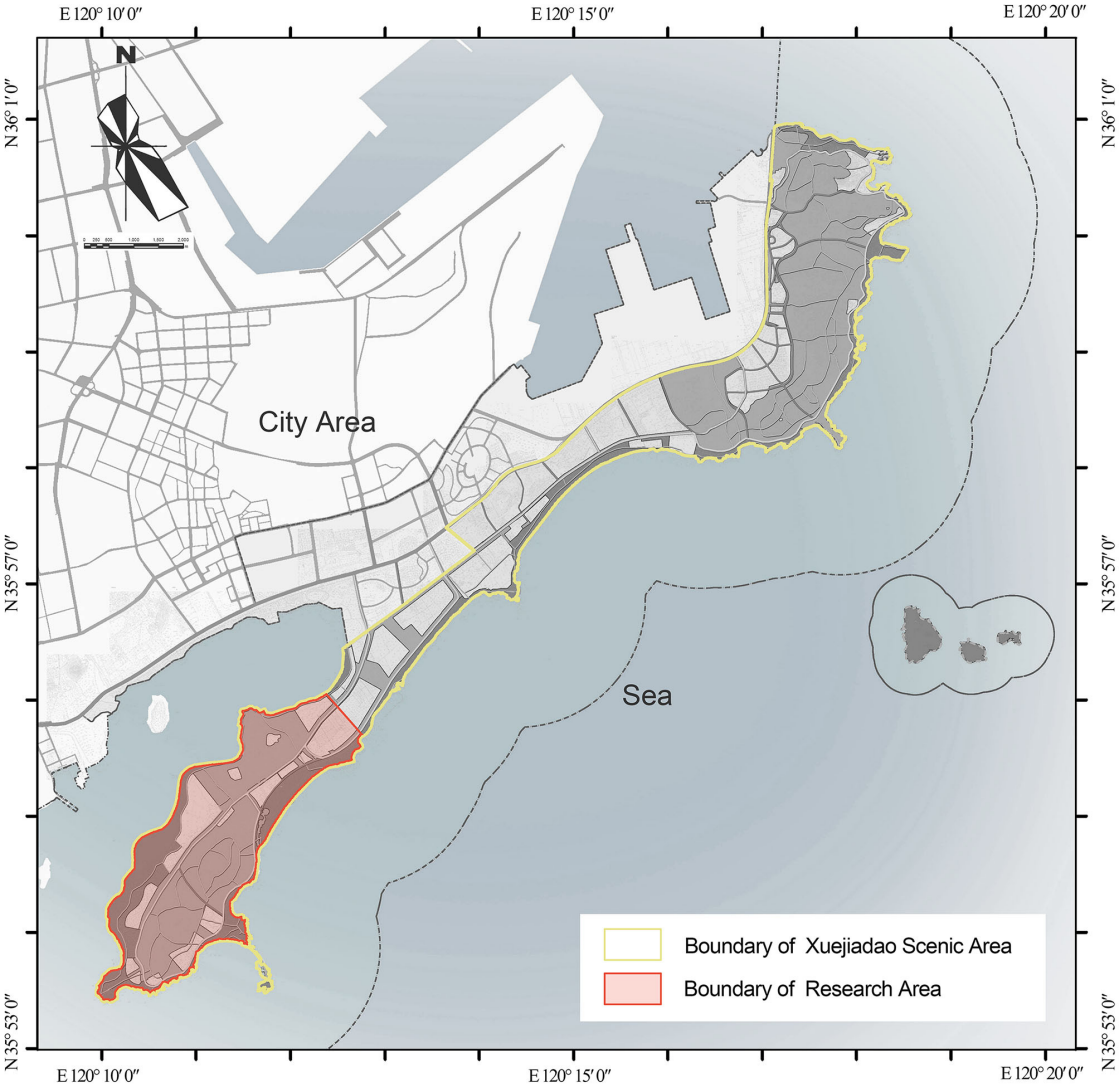
Geographic coordinate information and boundaries of the research area.

The research area is the west part of Xuejiadao Scenic Area with 837.83 hm2, located on the west coast of Qingdao, China. The geographic coordinate information and boundaries of the research area are shown in Figure 2. The whole Scenic Area is a long peninsula extending from northeast to southwest, connected to the land in the central. The length of the research area is about 6.2 km and the average width is about 1.6 km. No large rivers or lakes exist in this area. The annual average temperature is 12.2°C, −1.7°C in January for winter, and 27.3°C in August for summer. The recorded lowest temperature was −16.2°C (January 3, 1976), and the highest was 37.4°C (July 8, 1964). The average annual precipitation is 794.9 mm, and precipitation mainly occurs from June to September, accounting for 71.4% of the annual precipitation.

Based on Chinese vegetation regionalization, the research area belongs to warm temperate deciduous broadleaf forest region, Southern warm temperate deciduous Quercus Forest zone, Shandong peninsula Pinus densiflora, Quercus acutissima, and cultivated vegetation area. Because of the interference of human activities in history, the original natural forest was destructed. After decades of afforestation and conservation, shelterbelt was formed gradually with pine. The common woody plants are black pine, robinia, Quercus acutissima, black poplar, and others. Lespedeza bicolor and rosa multiflora are the majority of the bush, and the common herbs are Commelina communis L., Oenothera biennis, Chenopodium glaucum L., phragmites, Typha Orientalis, and others. There is one national protected wild plant species, Glycine soja. This area is ecologically sensitive and part of its land is restricted by ecological protection control line to protect environmental habitat values.

With an attractive natural landscape, the research area is close and convenient to the main district of the city. In the last decade, with the rapid expansion of the urban area, more and more construction works have appeared in this area. The local government has already realized the potential threat of this phenomenon to local ecology and landscape. Each new construction project in or near the scenic area needs to offer an assessment report before they get the licenses of construction. The report needs to analyze the impacts of a particular project on the surrounding ecological environment and landscape. Considering these problems, the west part of the Xuejiadao Scenic Area is chosen as the research area for this research.

### 3.2. Data Acquisition

**Figure 3:**
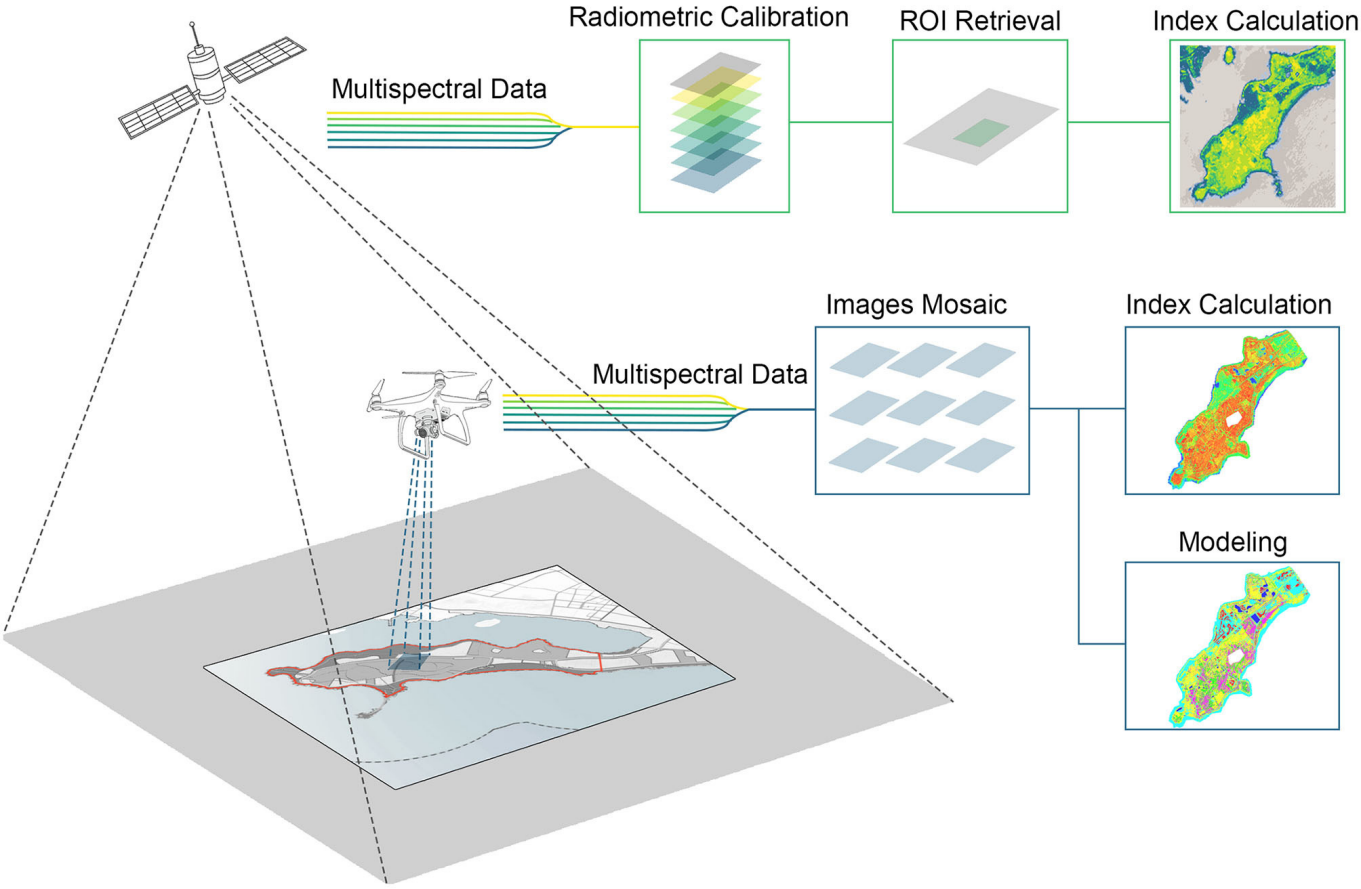
The process of remote sensing (RS) data acquisition.

Data acquisition is the initial stage of digital twins technology which is extremely critical. This is because the quality of data affects data modeling, vegetation recognition, result visualization, and decision support significantly. Satellite RS technique is highly developed in recent years and it can provide long time-series data, which benefits temporal analysis of vegetation change and human activities in the research area. In addition, considering the geographical size of the natural reserve is small, this study includes UAV RS to acquire ultra-high-resolution multispectral data in the research area. Figure 3 presents the process of data acquired by satellite and UAV.

#### 3.2.1. Satellite RS

Nowadays, the most common RS technique is implemented by earth-orbiting satellites, and it presents a high performance in multiple research fields such as land resource survey, forest fire prevention, city landscape transition, and vegetation investigation. Intentionally, this study focuses on the relationship between vegetation and urban development through recent years, so the RS data collected by Landsat series satellites are employed. Since these satellites are designed as sun-synchronous satellites, they can acquire multispectral images during moderate solar angle (25–30) in most areas of mid-latitudes of the Northern Hemisphere. These data are collected in very similar observation conditions, which is helpful to compare images in different years and benefits this study.

According to the administrative policy applied in the research area, this article mainly studies the regional enveloping since 2006, so RS data of both Landsat-7 and Landsat-8 can cover the period. Landsat-7 was launched in 1999 and carried an Enhanced Thematic Mapper sensor (ETM+). This sensor provides eight bands and most of them are with better resolution compared with previous satellites. In 2013, Landsat-8 was launched and carried advanced sensors, which are more accurate and comprehensive. The Operational Land Imager (OLI) and Thermal Infrared Sensor (TIRS) provide 11 bands with higher resolution. Table 1 shows the specification of multispectral sensors in Landsat-7 and Landsat-8. Considering the data are sensitive to atmosphere changing, cloudless images in summer and fall are retrieved to be analyzed.

**Table 1:** Specifications of Landsat-7 and Landsat-8.

**Landasat-7**			**Landasat-8**		
**Band name**	**Wavelehgth (μm)**	**Resolution (m)**	**Band name**	**Wavelehgth (μm)**	**Resolution (m)**
			1 Coastal	0.43–0.45	30
1 Bule	0.45–0.515	30	2 Blue	0.45–0.51	30
2 Green	0.525–0.605	30	3 Green	0.53–0.59	30
3 Red	0.63–0.69	30	4 Red	0.64–0.67	30
4 NIR	0.775–0.90	30	5 NIR	0.85–0.88	30
5 SWIR1	1.55–1.75	30	6 SWIR1	1.57–1.65	30
7 SWIR2	2.08–2.35	30	7 SWIR2	2.11–2.29	30
8 Pan	0.52–0.9	15	8 Pan	0.50–0.68	15
			9 Cirrus	1.36–1.38	30
6 TIR	10.4–12.5	60	10 TIRS1	10.6–11.19	100
			11 TIRS2	11.5–12.51	100

#### 3.2.2. Unmanned Aerial Vehicle Remote Sensing

**Figure 4:**
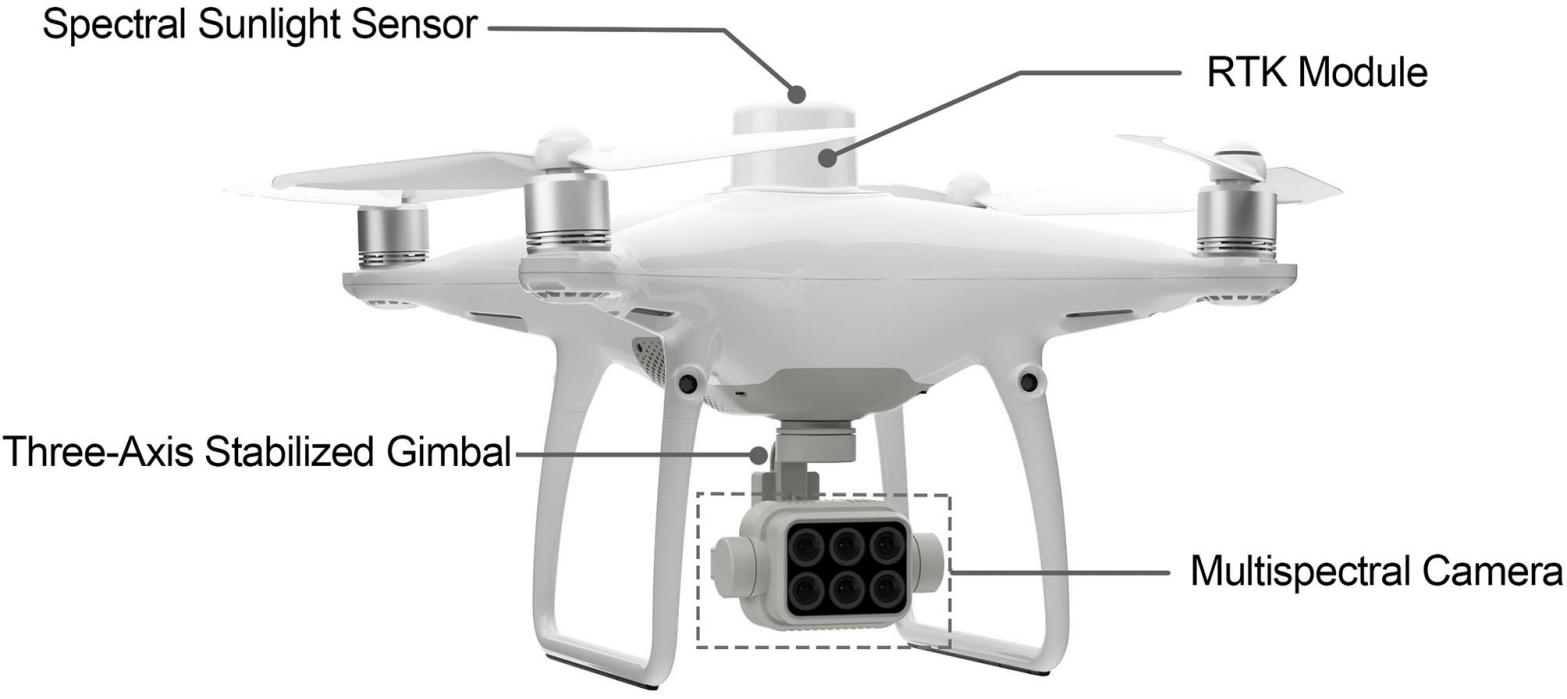
Features of P4M.

Unmanned Aerial Vehicle (UAV) technology is rapidly developed over the years and generates a large number of possibilities in various research areas. In 2019, DJI released the Phantom 4 Multispectral (P4M) drone which provides high usability and cost-efficient product to acquire ultra-high-resolution RS data. The P4M is based on the reliable Phantom 4 series drone and carries Real-Time Kinematic (RTK) module to reach centimeter-level precision. The camera with five narrow-band lenses and visible light is highly integrated into a three-axis stabilized gimbal with accurate calibration. These features allow users to collect high-quality data in small-scale research areas. Moreover, P4M integrates a spectral sunlight sensor on top of the drone capturing solar irradiance, which can be used to implement radiometric calibration automatically with post-processing software. Figure 4 shows the attractive features of P4M.

**Figure 5:**
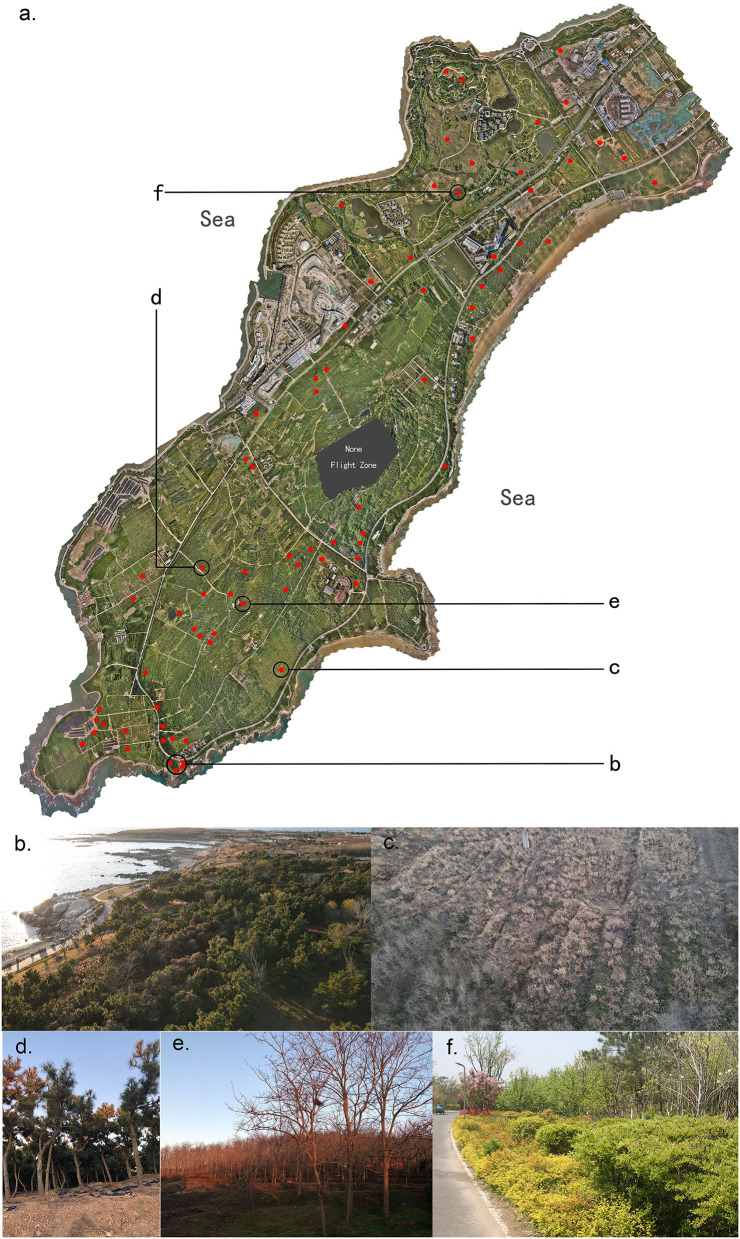
Locations of field investigation **(a)** and the images of vegetation **(b–f)**.

To balance the accuracy and efficiency of data acquisition, the relative altitude is set to 100 m; the front overlap ratio is set to 80%; the side overlap ratio is set to 70%; shutter interval is 2 s. According to the specification of P4M, the resolution under this setup is 5.3 cm per pixel, and the flight speed is 25.1 km per h. The total flight duration is 20 h and the flight distance is 460 km, which acquires 1,48,680 images occupying 505 GB storage. The resolution of each image including narrow bands and visible light is 1,600*1,300 pixels in the same, while the overall output with image mosaic is 80,038*91,959 per channel. Figure 5a presents the visible light result of RS data acquired by the P4M drone, and the Figures 5b–f are the images of local vegetation.

### 3.3. RS Index Calculation

There are various methods to utilize the multispectral data acquired by satellite and UAV, and the most effective way is index calculation. Many researchers have developed indices for different purposes. In order to identify water areas and analyze vegetation types in the research area, three RS indices are presented in this study. It should be noted that satellite RS data require the radiometric calibration process before index calculation, in order to acquire an accurate result. The general procedures can be done by using the following calculations:


(1) $$\begin{array}{r} L_{\lambda }=\left[\left(\frac{L_{max\lambda }-L_{min\lambda }}{DN_{max}-DN_{min}}\right)\times \left(DN-DN_{min}\right)\right]+L_{min\lambda } \end{array}$$

where,

$L_{\lambda }=spectral\;radiance\;at\;the\;sensor^{\prime }s\;aperture\;in\;\omega atts/m^{2}/$

*ster*/μ*m*,

*DN* = *quantized calibrated pixel value*,

*L*_*minλ*_ = *spectral radiance that is scaled to QCALMIN in ωatts*/

*m*^2^/*ster*/μ*m*,

*L*_*maxλ*_ = *spectral radiance that is scaled to QCALMAX in ωatts*/

*m*^2^/*ster*/μ*m*,

*DN*_*min*_ = *minimum quantized calibrated pixel value*

(*corresponding to L*_*minλ*_)*in DN*,and

*DN*_*max*_ = *maximum quantized calibrated pixel value*

(*corresponding to L*_*maxλ*_)*in DN*.

Additionally, the next procedure is to convert radiance to planetary reflectance, the calculation is:


(2) $$\begin{array}{r} \rho_{\rho }=\left[\frac{\pi \times \;L_{\lambda }\times \;d^{{}^{2}}}{ESUN_{\lambda }\times \;cos\theta_{s}}\right] \end{array}$$

where,

ρ_ρ_ = *unitless planetary reflectance*,

$L_{\lambda }=spectral\;randiance\;at\;the\;sensor^{\prime }s\;aperture$,

*d* = *earth*−*sun distance in astronomical units*,

*ESUN*_λ_ = *mean solar exo*−*atmospheric irradiance, and*

θ_*s*_ = *solar zenith angle in degrees*.

As a result, all the planetary reflectance of narrow bands can be retrieved and used for various index calculations.

Normalized Difference Vegetation Index is usually used for researching plants and forest-based on multispectral data. The value range of NDVI is from –1 to +1, where the smaller value indicates water and the larger value indicates dense vegetation. Since the chlorophyll in plants highly absorbs red light and reflects near-infrared, NDVI applies the following equation to indicate the growth situation:


(3) $$\begin{array}{r} NDVI=\left(\rho_{NIR}-\rho_{Red}\right)/\left(\rho_{NIR}+\rho_{Red}\right) \end{array}$$

Normalized Difference Vegetation Index presents a satisfactory performance in many RS scenarios, and it is easy to calculate. P4M drone can even provide real-time NDVI images during the flight, which benefits vegetation field survey.

Even though NDVI is commonly applied, the limitation cannot be ignored. Since it uses nonlinear mapping to stretch the value range, the low-value range is enhanced while the high-value range is compressed. Therefore, NDVI is much less sensitive in areas with high vegetation overcast and implement accurate identification. In consequence, this study includes GNDVI to evaluate water and nitrogen uptake into the plant canopy. GNDVI can improve the plant type recognition rate in dense vegetation areas, and the calculation can be expressed as follows:


(4) $$\begin{array}{r} GNDVI=\left(\rho_{NIR}-\rho_{Green}\right)/\left(\rho_{NIR}+\rho_{Green}\right) \end{array}$$

Moreover, GNDVI is related to Normalized Difference Water Index (NDWI) and can be used for water area extraction, because the calculation of NDWI is:


(5) $$\begin{array}{r} NDWI=\left(\rho_{Green}-\rho_{NIR}\right)/\left(\rho_{Green}+\rho_{NIR}\right) \end{array}$$

This means GNDVI is the negative NDWI, and the low value indicates water area.

### 3.4. Vegetation Identification Based on CNN

Convolutional Neural Network is a major branch of deep learning and be developed rapidly in multiple research areas, such as object detection, image segmentation, and image classification. Compared with the traditional fully connected neural network, CNN is based on local receptive fields theory and shared weights method to present efficient modeling. Since RS data are usually complicated and with ultra-resolution, the efficient modeling feature of CNN is highly suitable. There are various applications of CNN based on RS data, both in industrial and academic contexts. This study employees CNN modeling and traditional RS index methods to classify vegetation type in both satellite data and UAV data, and the performance analysis is presented.

There are four types of layers in CNN, which are input layer, convolution layer, pooling layer, and fully-connected layer. The input layer reads the multi-channel RS data to CNN, and each channel represents a narrow band. Since the narrow bands are sequential in the spectrum, 2-dimensional operation in the convolution layer can achieve satisfying performance in RS data modeling. The major purpose of the convolution layer is to use various kernels to extract and enhance features, thus improving model presentation ability. The pooling layer is another important component in CNN, which reduces the trainable parameters and moderates overfitting. This procedure is often presented by the max function, which reduces the resolution of input data. Therefore, the input data of the next convolution layer are reduced and benefit efficient modeling. The output of CNN is implemented by a fully connected layer with SoftMax function that integrates features from multi-channel and calculates the possibility of each class.

Considering the research area is a natural preserve, building construction is strictly controlled. Additionally, all the positions and layouts of buildings in the area can be acquired from the administration. As a result, it is straightforward to identify buildings in the RS data. Furthermore, the performance of NDWI is superb, water area can be extracted precisely. Besides, afforestation with single tree species is always harmful to nutrient cycling and soil fertility of forests (Bristow et al., 2006). It is also confirmed that planting coniferous trees mixed with broad-leaved trees will improve the soil nutrient and composition (Garau et al., 2019; Li et al., 2021). Measuring the proportion of different types of trees is important to understanding the ecology of an area. Hence, CNN is mainly employed to classify four types which are Bare land, grass, coniferous trees, and deciduous broad-leaved trees. In order to balance the accuracy and efficiency of modeling, the spatial resolution of original RS data acquired by P4M is resampled to 7,995*9,116, and then it is divided into images with 10*10 pixels. The total number of images in the research area is 2,72,618, while each image represents 28 m^2^. The data with true value is collected by field investigation and 10,040 images are identified, which is 3.68% of the total data. A total of 9,000 images are selected randomly as training datasets, and the other 1,040 images form test data for model evaluation. Table 2 describes the training data details.

**Table 2:** The training data details.

**Type**	**Number of samples**	**Percentage**
Bare soil	1,739	17.32
Grass	4,591	45.73
Coniferous trees	1,560	15.54
Deciduous trees	2,150	21.41

**Figure 6:**
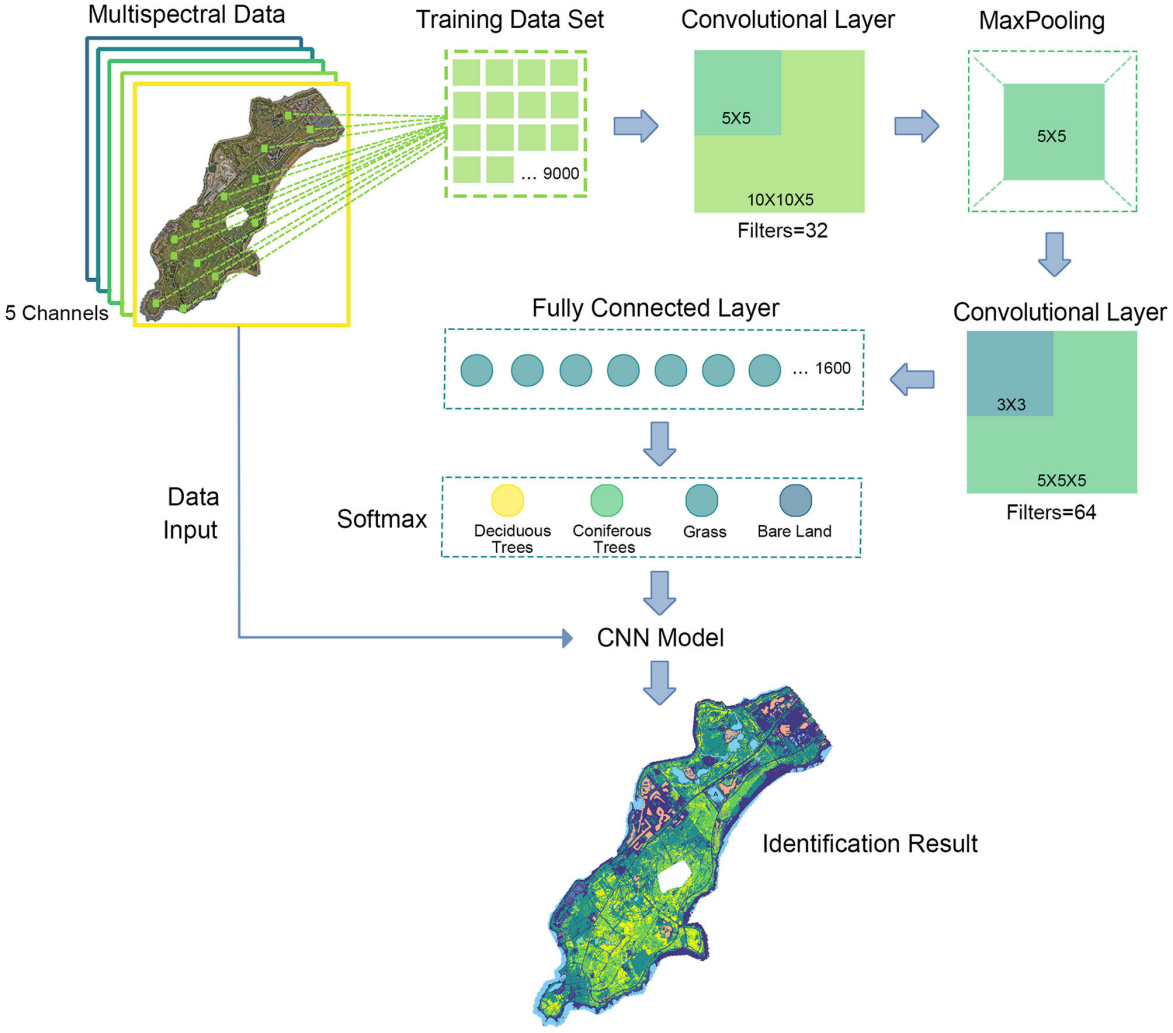
Convolutional Neural Network (CNN) modeling process.

The proposed CNN model architecture includes two convolution layers, one pooling layer, one fully connected layer, and one output layer. The input data is in 10*10 resolution with 5 channels. There are 32 filters in the first convolution and kernel size is set to 5*5. Then the max-pooling function reduces the resolution to 5*5 in each channel. The second convolution layer is with 64 filters in 5*5 resolution and then flatten to 1*1,600 nodes. The fully connected layer is performed as a tile structure with 1,600 nodes. Finally, the output layer with the SoftMax function classes each 10*10 image in the research area into one of four vegetation types. Figure 6 shows the vegetation type identification process based on CNN modeling.

## 4. Experimental Results and Discussion

In this section, the CNN modeling result is presented, and the comparison between CNN modeling and index classification is displayed. According to the result, satellite RS data is identified by the GNDVI method and contributed to the temporal analysis. Additionally, this research employs the CNN model for RS data acquired by the drone, supporting the spatial analysis. Based on the development and changes of plants over time, the changing characteristics and problems of plant coverage are summarized in temporal analysis. In addition, then the current plant situation of the research area is illustrated in spatial analysis.

### 4.1. Modeling Result

**Figure 7:**
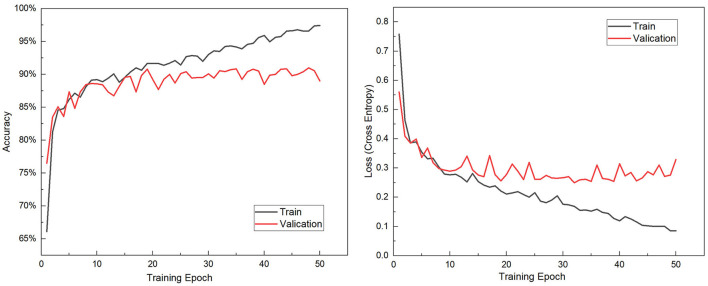
The accuracy and loss variance during the training epochs.

A workstation with Core i7-11700K CPU, 64GB RAM, and Geforce GTX 1050Ti GPU is employed to perform calculations. In the Windows 10 professional environment, DJI Terra software is in charge of drone data reconstruction while ENVI performs satellite data calibration. Python 3.8 and TensorFlow 2.7 are used for deep learning programming, which is the most popular framework in recent years. Following the CNN structure in Section 3, the model training can be implemented based on the training dataset. The training algorithm is Adaptive moment estimation (Adam), which is integrated into the TensorFlow optimizer and performs high efficiency with low memory requirement. Adam is regarded as the default training optimizer in common scenarios. Considering data size and efficiency, the training epochs are set to 50, and the batch size is 500. The validation dataset is split from the training dataset in 20%, and cross-entropy is employed as the loss function. Figure 7 shows the accuracy and loss variance during the training epochs. It shows that after 30 epochs, the CNN model is convergent and validation accuracy reaches 90.72. Then, training moves toward over-fitting, and validation accuracy begin fluctuating slightly. As a result, the CNN model with 30 epochs training is determined as the identification model. The model evaluation shows that the accuracy of test data is 91.24%, which means the model is appropriately trained and with great generalization. Furthermore, Table 3 presents the confusion matrix for each vegetation type, it shows great performance.

**Table 3:** The confusion matrix of CNN model.

**Identified as → **	**Bare soil**	**Grass**	**Coniferous trees**	**Deciduous trees**
Bare soil	167	10	0	0
Grass	5	455	4	0
Coniferous trees	0	22	192	17
Deciduous trees	0	13	20	135

Besides, index classification based on NDVI and GNDVI also proceeds in this study. By using the optimized threshold method, four classes are identified based on index value and the test dataset is applied for evaluation. The general accuracy of NDVI is 65.43% and GNDVI is 73.54%, both perform worse than the CNN model. Table 4 shows the comparison of precision and recall of three methods. It should be noticed that the precision of coniferous trees and deciduous trees is extremely low in the NDVI method. This is because NDVI is not sensitive in a high-value range and cannot distinguish them from each other. Moreover, according to the recall of coniferous trees, only 7.95% are correctly identified. GNDVI method achieves a better result, especially, improves recall of coniferous trees a lot, which states the advantage compared with NDVI method. Even though the performance of the CNN model is exciting, it requires RS data in ultra-resolution and high-quality training datasets, which limits the universality. As a result, this study employs the CNN model for RS data acquired by the drone, while satellite RS data is identified by the GNDVI method.

**Table 4:** The comparison of precision and recall of three methods.

**Vegetation type**	**NDVI (65.43%)**		**GNDVI (73.54%)**		**CNN (91.24%)**	
	**Precision**	**Recall**	**Precision**	**Recall**	**Precision**	**Recall**
Bare soil	83.52%	83.32%	97.54%	86.60%	97.09%	94.35%
Grass	78.94%	74.71%	86.53%	80.98%	90.98%	98.06%
Coniferous trees	34.44%	7.95%	43.11%	39.68%	88.89%	83.12%
Deciduous trees	44.45%	72.92%	55.88%	71.66%	88.82%	80.36%

### 4.2. Temporal Analysis

**Figure 8:**
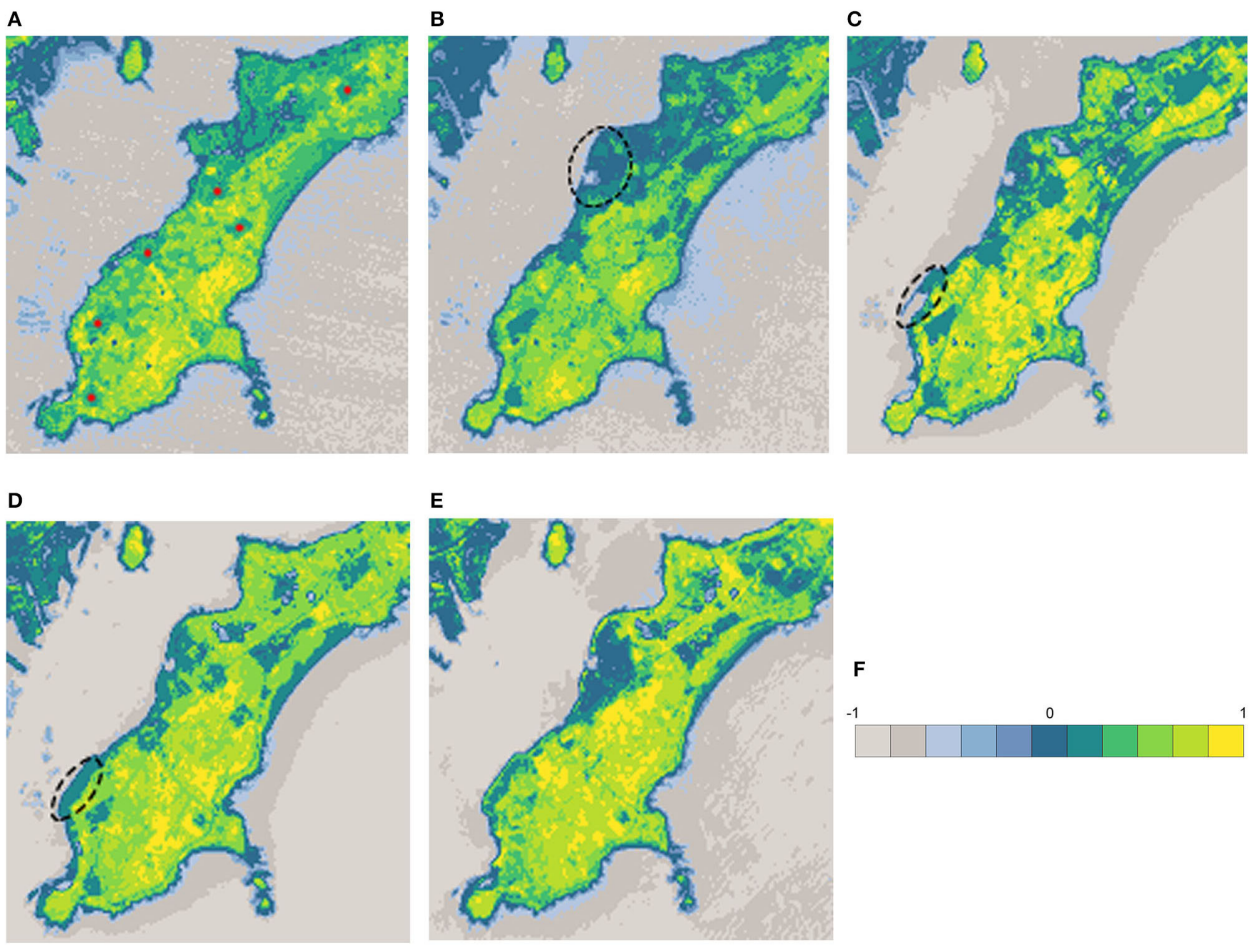
Landsat GNDVI results of years. **(A)** 2006.10, **(B)** 2010.10, **(C)** 2013.8, **(D)** 2017.9, **(E)** 2020.6, and **(F)** Legend.

The annual amount and distribution trend in land coverage is determined through the GNDVI images from Landsat. The study period is from 2006 to 2020, and take every 3 to 4 years as a time interval for observation and analysis, since the changes were more obvious. The GNDVI results were shown in Figure 8. The land covered by plants was presented in colors from yellow to green, and yellow was recognized as trees and green as grass.

**Figure 9:**
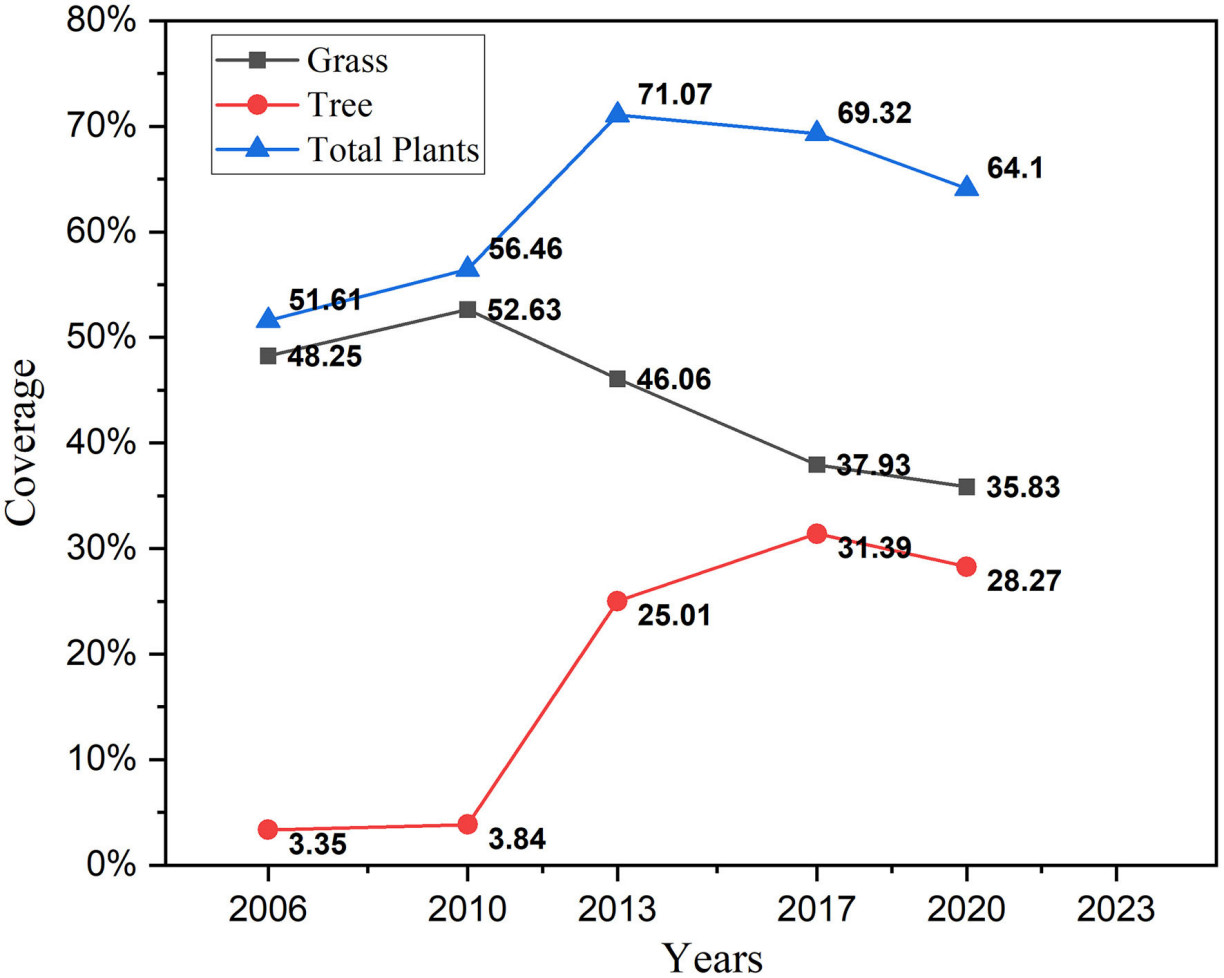
The variation trend of vegetation types.

Figure 9 summarizes changes in the grass, tree, and total plants coverage by analyzing the GNDVI images in Figure 8. In 2006, there were 6 villages in the research area, which was shown in Figure 8A. A total of 51.61% of the land was covered by plants, the majority of which was agriculture. The GNDVI values of the whole research area were low, which meant the quality of plant coverage was at a low level. In 2010, under the requirement of urban sprawl and government policies, a large proportion of agriculture activities had been stopped. The plant coverage grew up to 56.46%, even though sea reclamation had enlarged the area of land (Figure 8B). The quality of vegetation was still at a low level as grass took the majority. The proportion of trees was increased dramatically in 2013 because of the cultivation of economic forests in a large area. Under the circumstance of sea reclamation (Figure 8C), the plant coverage rose obviously to 71.07%. The proportion of grass declined to 46.06%, mainly because of the transformation to the tree. Because of new construction works and sea reclamation (Figure 8D), the coverage of the plant fell to 69.32% and the reduction totally happened on grass. While the proportion of trees kept growing up to 31.39%. In 2020, the remaining buildings in these villages had been demolished and grass grew naturally blurring the boundaries of the villages. However, more loss of plant coverage happened. A 3.12% reduction was taken by trees and 2.15% by grass.

### 4.3. Spatial Analysis

**Figure 10:**
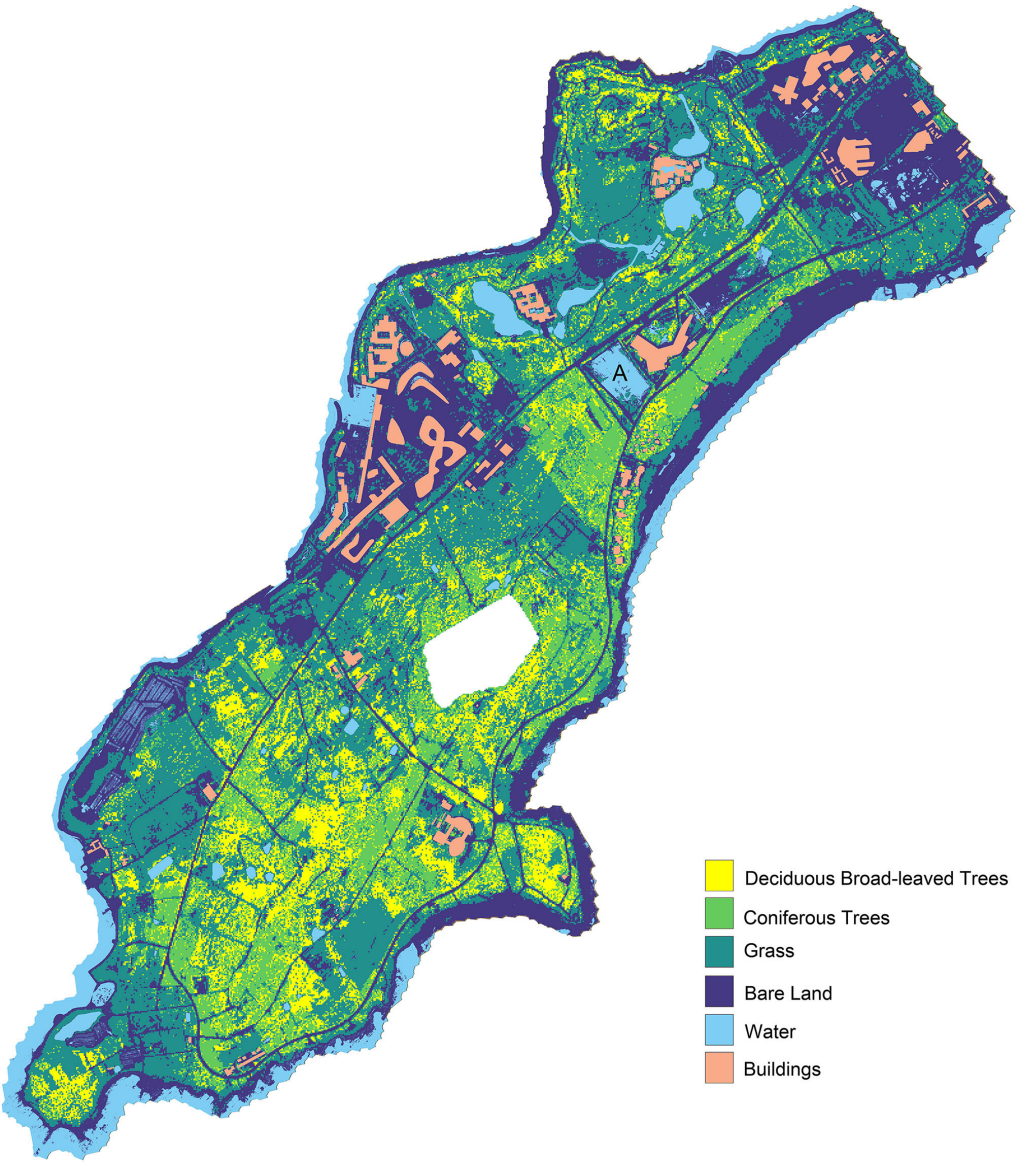
The coverage and distribution of land cover categories.

**Figure 11:**
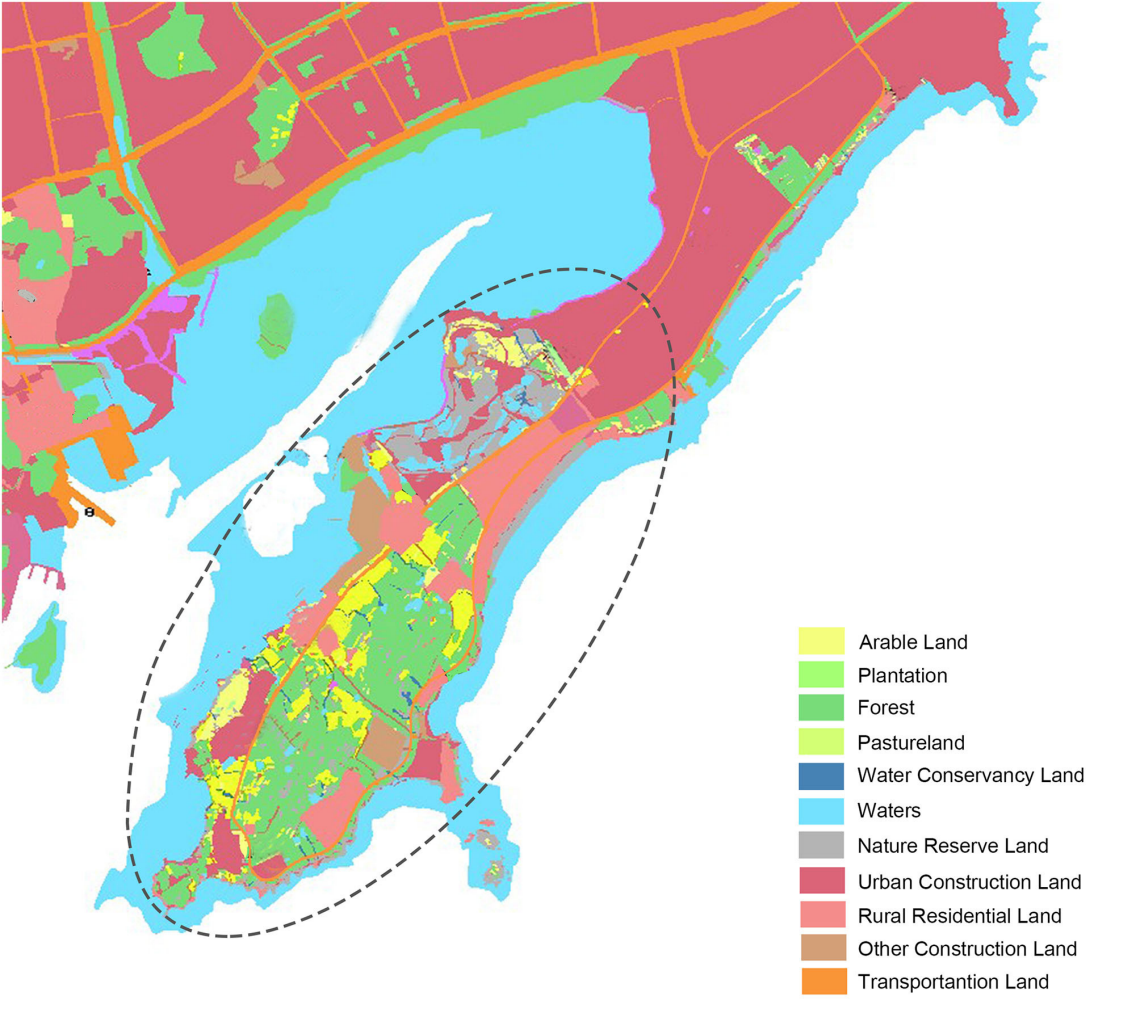
Master planning of land use (2006–2020).

The coverages of 6 land cover categories are demonstrated in Figure 10. Plants dominate the research area, and the coverage is 62.63% contenting grass (37.61%), coniferous trees (12.93%), and deciduous broad-leaved trees (12.09%). The south and southeast of the area have considerable forest coverage, but there are a lot of bare lands and grassland interspersed within the forest range. The continuity of forests is in poor condition. Grass occupies more than 50% of plant coverage, mainly distributed in the National Wetland Reserve on the north and the village demolition sites. The bare land is 25.86%, ranking in the second, mainly distributed in and around the construction area. There are two intensive construction areas located on the north and the northwest with a large proportion of total research land area. The building takes 3.70% of land coverage, and completed buildings, small scale in the majority, are scattered around the coastal side with considerable plants surrounding by. Water (7.81%) is distributed throughout the area in the form of ponds, and more than half of it is located on the National Wetland Reserve on the north. Compared with the Master planning of land use (Figure 11), the present spatial distribution is basically consistent with it.

The National Wetland Reserve is established since the year 2013. For now, the vegetation coverage of it is 76.1%, including 60.7% of grass, 15.4% of trees. In this wetland reserve, the majority of trees are planted and part of the artificial landscape. The large area of bare land (10.8%) is still existing and buildings take 4.7% area of this wetland reserve. The situation of ecology is not optimistic, and there is a risk of vegetation degradation and soil erosion. In addition, based on the Master planning, the National Wetland Park is surrounded by a large amount of urban construction land and rural residential land without transitional space. The nature reserve land is intersected by urban construction land, leading to the fragmentation of the landscape. Extensive construction and human activities may exacerbate the potential risks and impede the conservation and regeneration of ecology.

Four of the village demolition sites have been abandoned for more than 3 years without construction, which are mainly covered by naturally grown grass and bare land. In terms of the land area increased by sea reclamation, most of them are bare and others are covered by grass. Water-worn ravines have formed on the surface of these areas, which means soil erosion has already happened. Based on the Master planning, these areas belong to construction land, and it is hard to predict when these sites will start construction activities. There is a risk of deterioration without appropriate arrangements.

For the sites under construction, most of the vegetation has been destroyed. According to the Master planning, vacant lands around these sites are also affected, used to store construction materials or build temporary housing for construction workers. Most surfaces of these areas are bare. Even though the research area is only under urban construction for about 10 years, unduly completed projects and abandoned buildings have existed for several years. Site A (Figure 10) has been developed and constructed, but it has been abandoned before the completion of construction. Now, the site has become a standing water pond.

## 5. Discussion

It can be drawn from the spatial and temporal analysis that human activities have a greatly decisive influence on the quality and quantity of plant coverage. Before 2017, planting economic forest and the policies carried out by local government played a significant role in the conservation and regeneration of local vegetation. However, degeneration appeared in 2017 mainly because of construction activities. Such operation of restoring and then destroying is confused and debatable. The economic forests only have been planted for less than 10 years with pine and cypress, which are relatively simple species. The biodiversity conservation and ecological values of them are far less than primeval forest (Bremer and Farley, 2010; Gibson et al., 2011). As a scenic area, the ecological environment of the research area is still very sensitive and fragile, and it needs more time for conservation and regeneration. In addition, the unduly completed projects and bare land for many years have reflected the incompetent management of relevant authorities. Under the circumstance of inevitable urbanization, it is the construction activities that will have continually great impacts on the plant coverage of this area in the future. Thus, effective management and detailed control planning strategies are needed.

Combined with the natural and social situation of the region, 6 suggestions are summarized based on the research results:

1. The Master planning of land use needs to be adjusted effectively according to the current situation and urban actual development. Especially in the area of the National Wetland Reserve, it is essential to reduce the amount of urban construction land, enhancing the integrality of wetland landscape for better conservation.
2. Appropriate management strategies are the most effective method to avoiding excessive construction of buildings and the destruction of vegetation. The sequence of construction land should be carefully arranged, and the village demolition sites can be considered to develop in priority. Besides, unduly completed projects and abandoned buildings need to be re-planned and constructed.
3. Limit the total area of land under construction at the same time, to avoid a large amount of loss occurring on vegetation in a short period. In addition, the destruction of vegetation outside construction sites should be strictly prohibited.
4. Effective greening measures should be taken for sites that will not be developed and constructed in the short term, such as being taken as temporary economic arboriculture gardens and spreading seeds on bare ground.
5. The vegetation coverage of the whole area should be strictly controlled to guarantee the minimum vegetation coverage and basic ecological stability. In addition, except for the greening rate, other greening indicators of the construction projects in the Scenic Area should be specific required, such as rate of green roofs and proportion of trees.
6. Gradually increase the species of native plants that adapted to the climate and living conditions of the research area, increasing biodiversity and enhancing ecological stability.

## 6. Conclusion

This study aims to research the relationship between urban expansion and the natural environment for the last 15 years by identifying different vegetation types in the research area. The technique of digital twins is used in the whole research process. The multispectral data of the research area are collected by Landsat series satellite and UAV. NDVI, GNDVI, and CNN modeling are used for identification. Additionally, a comparison of precision and recall of three methods is made, with the result that the CNN model has the highest accuracy (91.24%) for RS data and acquired by drone, while satellite RS data is identified by the GNDVI method. Based on the identification results, spatial-temporal analysis of the research area is supported, and the characteristics of land cover change and current problems are summarized. Under the circumstance of urbanization for the last 15 years, the incompetent management, unreasonable planning, and short period for conservation and regeneration are the essential problems of the research area. As a natural preserve area, the ecological environment of the research area is still very sensitive and fragile, and it needs more time for conservation and regeneration. In addition, it is the construction activities that will have continually great impacts on the plant coverage of this area in the future. The 6 suggestions that have been summarized above may be helpful to effective management and planning strategies.

This study has provided a good reference case for related research, but improvement is still needed in data collection and data modeling. The resolution of satellite images from Landsat is low with insufficient details, so the accuracy of vegetation recognition is low. Thus, it is not quite appropriate to the research area on a small scale. The UAV image acquisition efficiency is unsatisfactory, which is not suitable for the research with a long research period that requires multiple image acquisition. The CNN model used in this research is a simple and optimized model that can be used to improve the accuracy of identification. In addition, since it cannot go back to the past for field investigation, the CNN model has limitations in the identification of historical data.

## Data Availability Statement

The raw data supporting the conclusions of this article will be made available by the authors, without undue reservation.

## Author Contributions

WG and XW contributed to conception and design of the study. DZ and XL collected the database. XL performed the data preprocessing and index calculation. XS performed the CNN modeling. DZ and XS contributed to visualization. DZ and XW analyzed and interpreted the results. DZ and XL wrote the manuscript. All authors reviewed the results and approved the final version of the manuscript.

## Conflict of Interest

The authors declare that the research was conducted in the absence of any commercial or financial relationships that could be construed as a potential conflict of interest.

## Publisher's Note

All claims expressed in this article are solely those of the authors and do not necessarily represent those of their affiliated organizations, or those of the publisher, the editors and the reviewers. Any product that may be evaluated in this article, or claim that may be made by its manufacturer, is not guaranteed or endorsed by the publisher.
