# Supplementary material for: Applying Digital Twins to Research the Relationship Between Urban Expansion and Vegetation Coverage: A Case Study of Natural Preserve
Source: Front Plant Sci. 2022 Feb 15;13:840471. doi: 10.3389/fpls.2022.840471 (PMC8885539; doi:10.3389/fpls.2022.840471)

Experimental environment

TensorFlow '2.7.0'

Python 3.8.8

CPU: i7-11700K

RAM：64.0 GB

Windows 10 64-bit, professional

1. Image: 9116*7995 pixels
2. Sample size: 10*10 pixels
3. Number of dataset: 10040; Training: 9000; Test: 1040
4. Input channels: narrow red, narrow green, narrow blue, NIR, red edge, visible red, visible green, and visible blue.


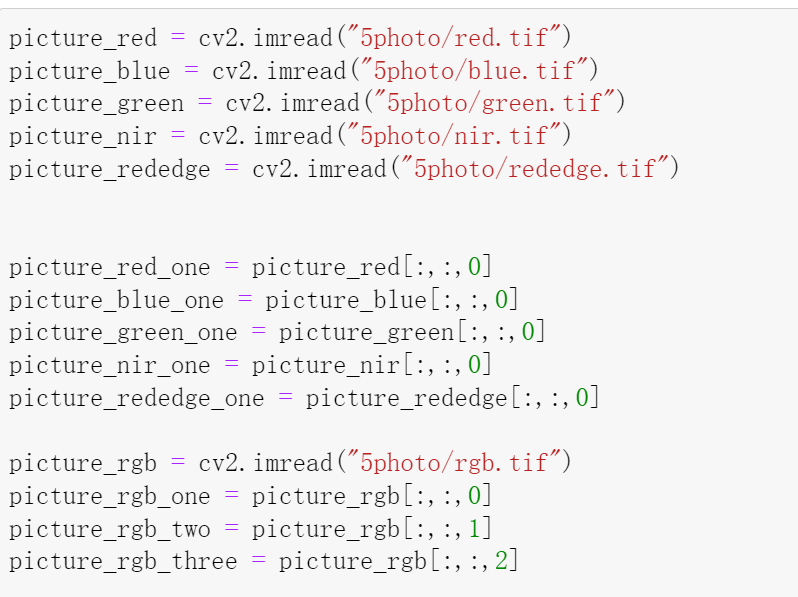


1. CNN model structure


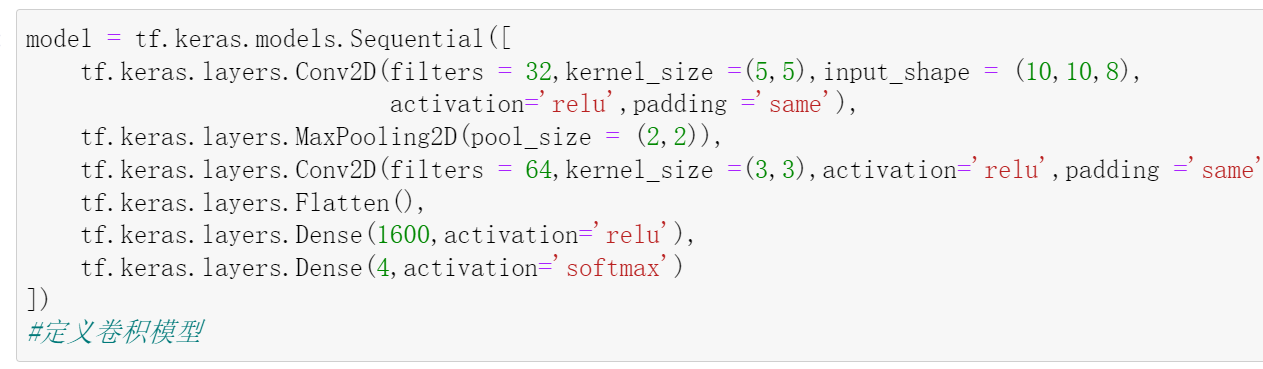


1. Training epochs=30

batch_size=500

validation_split=0.2

1. Result

Training:

loss: 0.0944

accuracy: 0.9665

val_loss:0.1725

val_accuracy:

Test:

loss:0.1881

accuracy:0.9346


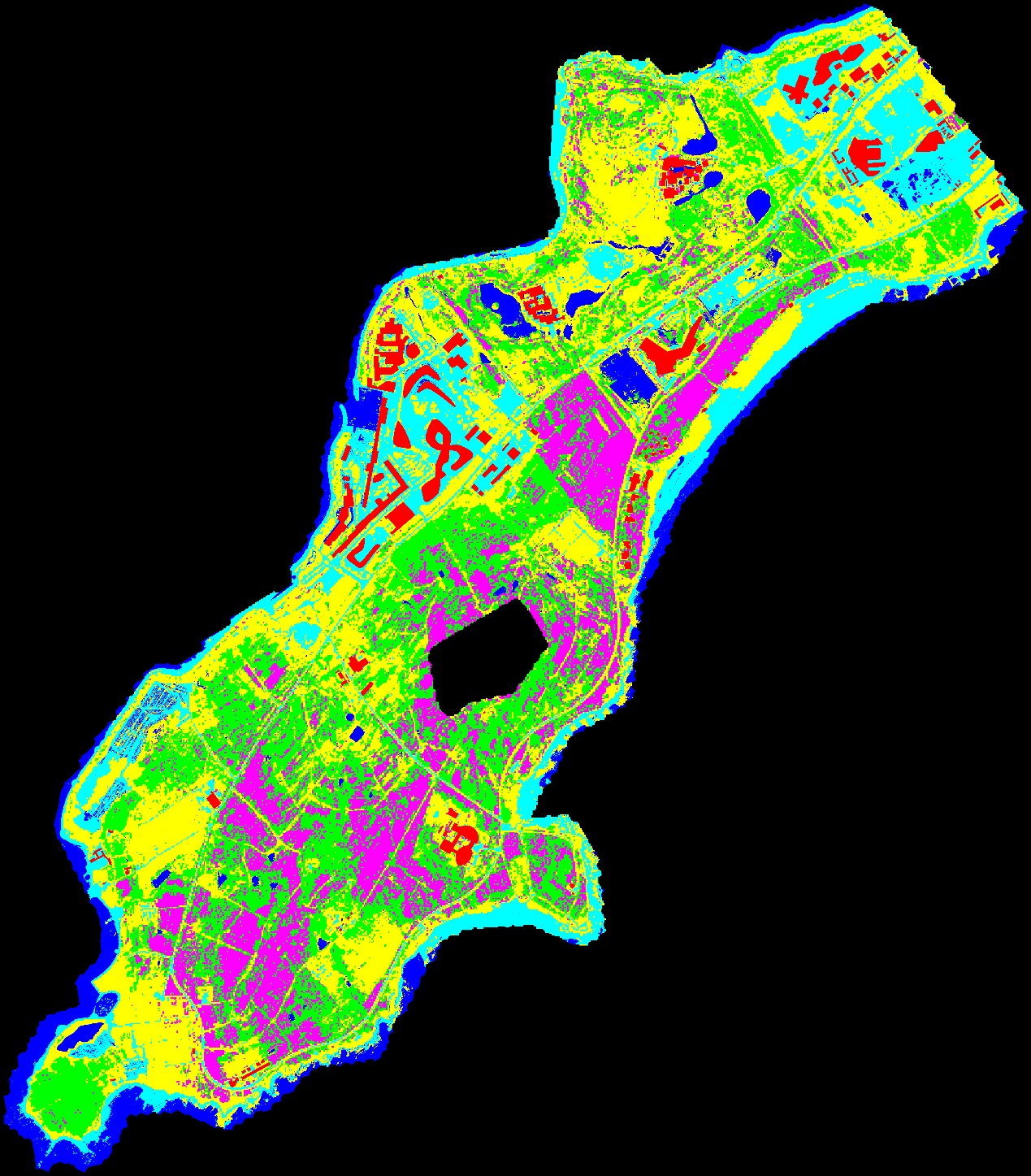


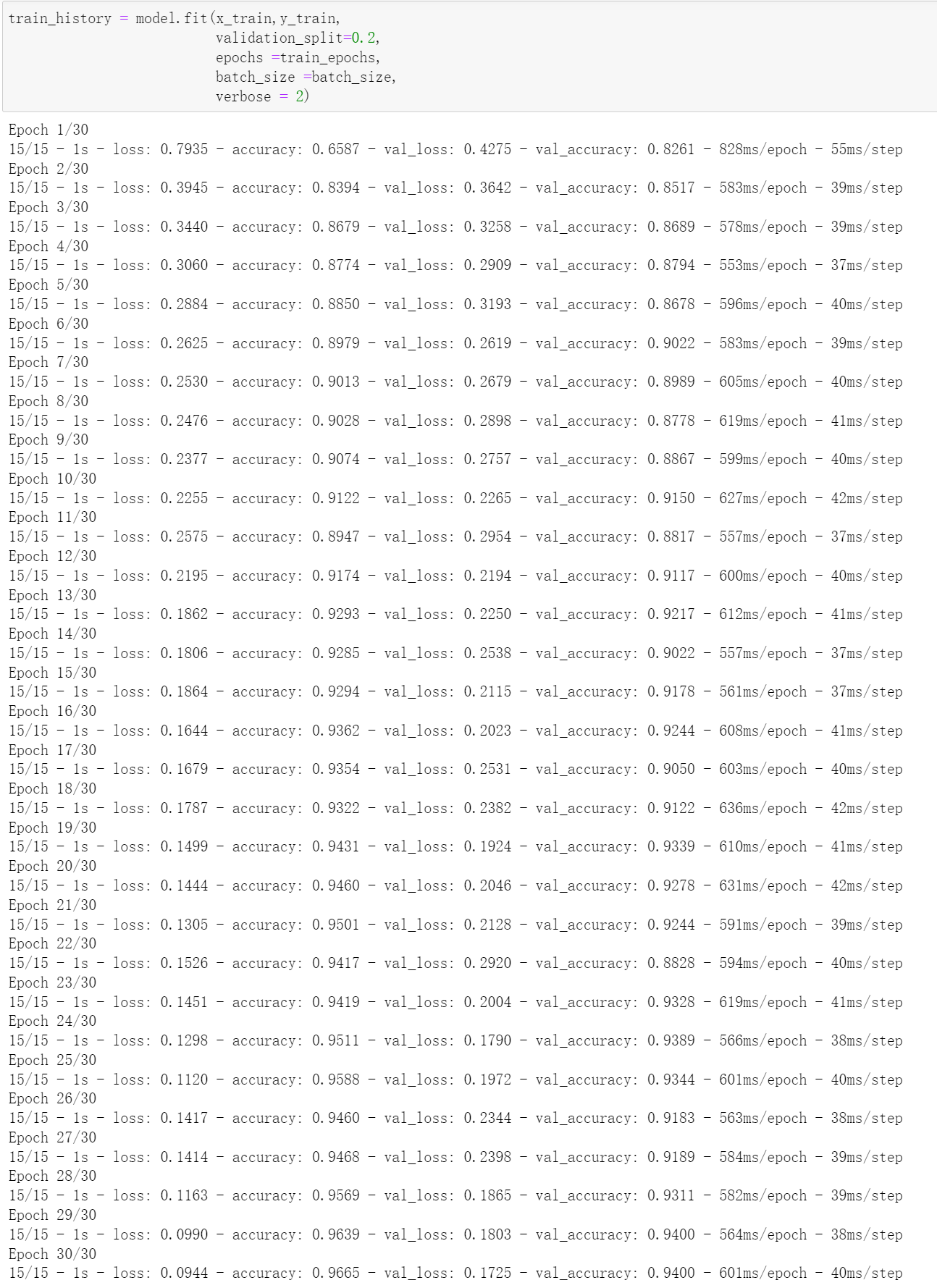


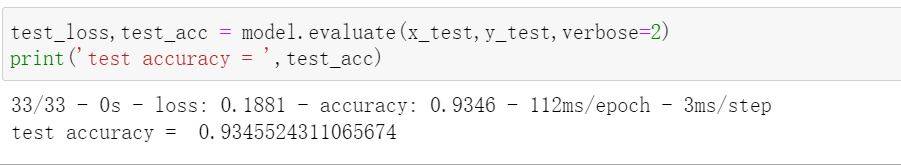


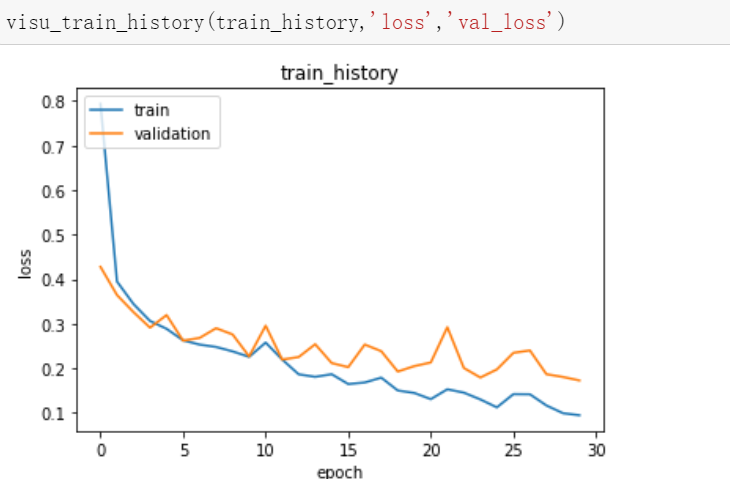


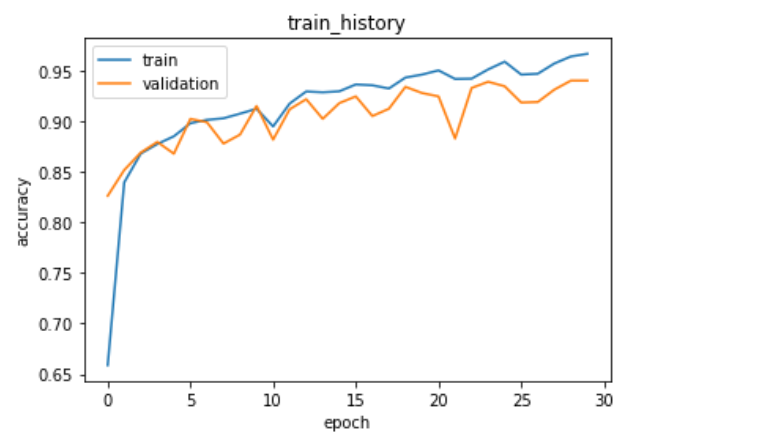


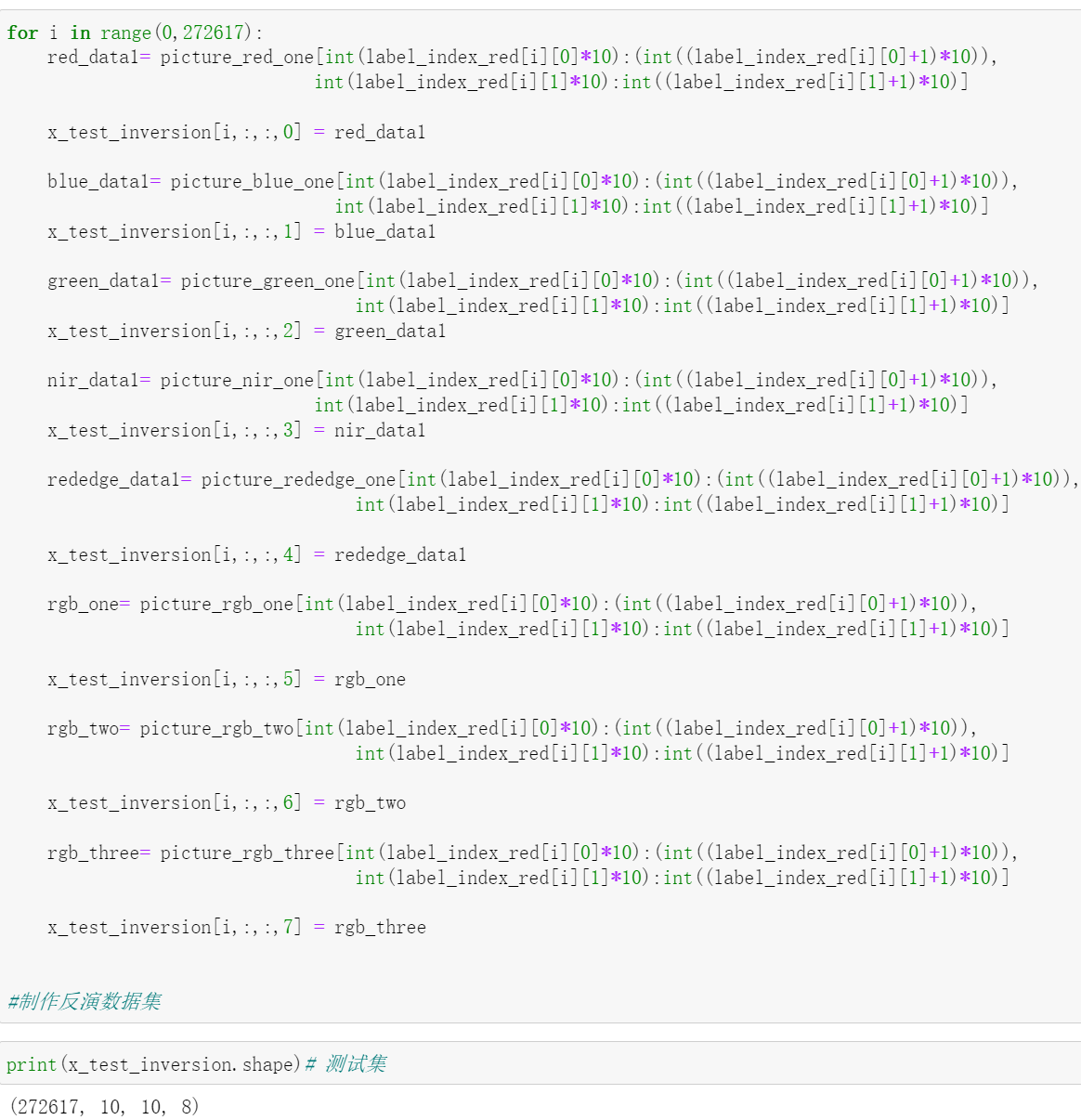


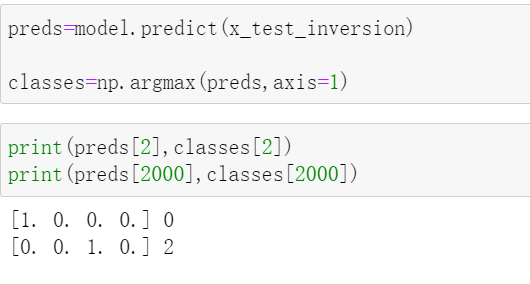

Supplement: Supplementary file 1 [file Data_Sheet_1.docx]
